# Genome-wide analysis of Myo-inositol oxygenase gene family in tomato reveals their involvement in ascorbic acid accumulation

**DOI:** 10.1186/s12864-020-6708-8

**Published:** 2020-04-06

**Authors:** Shoaib Munir, Muhammad Ali Mumtaz, John Kojo Ahiakpa, Genzhong Liu, Weifang Chen, Guolin Zhou, Wei Zheng, Zhibiao Ye, Yuyang Zhang

**Affiliations:** 1 Key Laboratory of Horticultural Plant Biology, Ministry of Education, Huazhong Agricultural University, Wuhan, 430070 China; 2 Wuhan Academy of Agricultural Sciences, Wuhan, 430065 China; 3 HZAU Chuwei Institute of Advanced Seeds, Wuhan, 430070 China

**Keywords:** Tomato (*Solanum lycopersicum*), MIOX, Myo inositol, AsA, Oxidative stress

## Abstract

**Background:**

Ascorbic acid (Vitamin C, AsA) is an antioxidant metabolite involved in plant development and environmental stimuli. AsA biosynthesis has been well studied in plants, and MIOX is a critical enzyme in plants AsA biosynthesis pathway. However, Myo-inositol oxygenase (MIOX) gene family members and their involvement in AsA biosynthesis and response to abiotic stress remain unclear.

**Results:**

In this study, five tomato genes encoding MIOX proteins and possessing MIOX motifs were identified. Structural analysis and distribution mapping showed that 5 MIOX genes contain different intron/exon patterns and unevenly distributed among four chromosomes. Besides, expression analyses indicated the remarkable expression of *SlMIOX* genes in different plant tissues. Furthermore, transgenic lines were obtained by over-expression of the *MIOX4* gene in tomato. The overexpression lines showed a significant increase in total ascorbate in leaves and red fruits compared to control. Expression analysis revealed that increased accumulation of AsA in *MIOX4* overexpression lines is possible as a consequence of the multiple genes involved in AsA biosynthesis. Myo inositol (MI) feeding in leaf and fruit implied that the Myo-inositol pathway improved the AsA biosynthesis in leaves and fruits. *MIOX4* overexpression lines exhibited a better light response, abiotic stress tolerance, and AsA biosynthesis capacity.

**Conclusions:**

These results showed that *MIOX4* transgenic lines contribute to AsA biosynthesis, evident as better light response and improved oxidative stress tolerance. This study provides the first comprehensive analysis of *the MIOX* gene family and their involvement in ascorbate biosynthesis in tomato.

## Background

Ascorbic acid (Vitamin C, AsA) is a ubiquitous metabolite in plants which is involved in resistance to environmental stresses. It mainly protects cells from oxidative damage by scavenging reactive oxygen species (ROS, e.g., superoxide and H_2_O_2_) produced by environmental stresses such as cold, drought, and pollutants [1]. AsA biosynthesis in plants occurs naturally through a pathway with d-mannose and l-galactose as intermediates [2]. It is also an essential component of human nutrition. However, humans and other animal species cannot synthesize AsA due to a mutation in the last enzyme required in the AsA biosynthesis pathway (L -gulono-1,4-lactone oxidase or GLOase). Humans obtain AsA (vitamin C) regularly from dietary sources like fruits and vegetables, generating great scientific interest in AsA biosynthesis and regulation in plants [3]. The discovery of plant AsA biosynthesis pathways makes it possible to increase its content in plants by regulating the expression of genes involved in this pathway for improved nutritive values and enhanced tolerance to environmental stresses. Animals utilize D-glucuronate as a precursor for vitamin C formation. However, plants rely on different alternate routes for AsA synthesis like the D-mannose/L-galactose (Man/Gal), L-gulose, D-galacturonate (GalU), and Myo-inositol pathways [4].

Myo-inositol (MI) is a unique molecule that plays an essential role in the growth and development of eukaryotic cells [5]. MI is also involved in different processes, including phosphatidylinositol (PtdIns) signaling pathway, cell wall biosynthesis, phytic acid biosynthesis, auxin storage, and transport, and the production of stress-related molecules [6]. In plants, MI is one of the most abundant forms of inositol [7] synthesized from glucose by the enzymes, L-myo-inositol 1-phosphate synthase (MIPS), and inositol monophosphate phosphatase (IMPase) [6]. The MI oxidation pathway (MIOP) consumes MI and considered a critical route for cell wall polysaccharide synthesis [5]. Along this pathway, Myo-inositol oxygenase (MIOX) as an essential monooxygenase enzyme, catalyzes the transferring process of MI into D-glucuronic acid (D-GlcUA). The UDP-GlcA is a vital sugar precursor for plant cell walls [8] and plays a significant role in the production of hemicellulose precursors [9]. MIOX maintains myo-inositol content necessary for the synthesis of some low molecular weight compounds in plants [10]. MIOX is a critical enzyme in plants AsA biosynthesis pathway. A previous study has proposed that MIOX gene overexpression leads to a 2- to 3-fold increase in AsA content of transgenic *Arabidopsis* plants [11]. However, recent studies have shown that MIOX controls the level of *Myo*-inositol without increasing AsA content [12, 13], which was further confirmed in rice where *OsMIOX* expression was induced by drought overexpression lines evident in improved drought tolerance in rice, without altering the level of AsA before and after stress [14]. On the contrary, *AtMIOX4 (Arabidopsis)* has been reported to play a significant role in AsA biosynthesis using *Myo*-inositol as a precursor [15]. Additionally, abiotic stress tolerance was observed in the overexpressing line by an increase in ascorbic acid content [1]. These findings indicate that MIOX is crucial in abiotic stress tolerance and might be activated under sugar starvation conditions to generate alternative sugar sources, thereby indirectly contributing to metabolic homeostasis. However, MIOX involvement in AsA biosynthesis in plants remains unclear.

In this study, we performed a genome-wide identification of the MIOX gene family and investigated the role of *Myo*-inositol (MI) as a precursor of AsA biosynthesis in tomato. *MIOX4* transgenic lines resistance to abiotic stresses was also studied.

## Results

### Identification, sequence alignment and phylogenetic analysis of tomato MIOX genes

Four Arabidopsis MIOX protein sequences were downloaded from the TAIR database (https://www.arabidopsis.org) as queries for homologous sequences in the tomato genome database (http://solgenomics.net/) as described in methods. Keywords like ‘myo-inositol oxygenase’ and ‘MIOX’ were used to do a homologous search in the tomato database. All the retrieved sequences were aligned to remove redundant sequences after a similarity comparison. Based on domain analysis as described in the methods, a total of 5 protein sequences were identified as unique MIOX proteins in tomato, which contains MIOX motifs or MIOX domains (IPR007828, and PF05153). The nucleotide sequences, amino acids, theoretical isoelectric point (pi), molecular weight (Mw), sequence length (aa), and other relevant information were obtained from the SGN or ExPASy-ProtParam database (Table 1). *MIOX2* contains the maximum number of 1053 bp of nucleotides and 350 amino acids with 40.87 kDa molecular weight and 5.68 pI. While *MIOX3* contains a minimum number of  756 bp of nucleotides and 251 aa with 28.87 kDa Mw and 4.99 pI; *MIOX1*, *MIOX4*, and *MIOX5* contain 999, 954 and 870 bp of nucleotides and 332, 317 and 289 aa, respectively. The MIOX five members were 66–80% identical at the amino acid level. MIOX genes were localized mostly in the cytosol and nuclear along with other cell organelles such as endoplasmic reticulum, vacuolar membrane, chloroplast, mitochondria, cytoskeleton, and plasma membrane (Table 1).

**Table 1:** Characteristics of MIOX gene family in tomato

Gene name	Annotated locus-1	Unigene Number	Chr-2	CDS-3	AA-4	Pls-5	MW (kDa)-6	Localization predicted by PSORT-7	Location	Strand
*Sl*MIOX1	Solyc06g062430	SGN-U588655	6	999	332	5.03	38.53	cyto: 7, nucl: 3, chlo: 2, mito: 1	SL4.0ch06:37032305..37035813	–
*Sl*MIOX2	Solyc10g005400	SGN-U588654	10	1053	350	5.68	40.87	chlo: 6, mito: 4.5, cyto_mito: 3, nucl: 2	SL4.0ch10:189928..191479	–
*Sl*MIOX3	Solyc11g006570	–	11	756	251	4.99	28.87	cyto: 8.5, cyto_E.R.: 5, nucl: 3, cysk: 1	SL4.0ch11:1245801..1247489	–
*Sl*MIOX4	Solyc12g008650	SGN-U565448	12	954	317	5.09	37.13	cysk: 8, cyto: 4, nucl: 1	SL4.0ch12:2075407..2079388	–
*Sl*MIOX5	Solyc12g098120	SGN-U588652	12	870	289	5.51	34.09	cyto: 8, nucl: 4, pero: 1	SL4.0ch12:65291322..65292191	+

1. The International Tomato Annotation Group (ITAG) annotates number

2. Chromosome number in which the gene resides

3. Length of the coding region in base pairs

4. Number of amino acids of the deduced amino acid sequence

5. PIs, theoretical isoelectric point

6. MW, molecular weight, Kda

7. PSORT: https://www.genscript.com/tools/wolf-psort; *Cyto* cytosol; *ER* endoplasmic reticulum; *Vacu* vacuolar membrane; *Chlo* chloroplast; *Nucl* nuclear; *Extr* extracellular; *Mito* mitochondria; *Cysk* cytoskeleton; *Plas* plasma membrane; −, not named

**Fig. 1:**
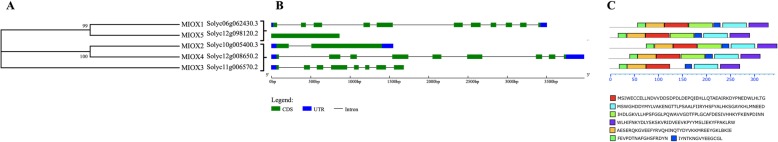
Phylogenetic relationships, gene structure, and motif compositions of tomato MIOX genes. **a**. Multiple alignments of 5 MIOX proteins from tomato were conducted using ClustalX2.1, and the phylogenetic tree was constructed using MEGA5.0 with 1000 bootstrap replicates. These 5 *SlMIOX* were clustered into two groups. **b**. Exon/intron organization of tomato MIOX genes using GSDS. The green boxes represent exons and the black lines represent introns. The blue boxes represent UTR regions. The sizes of the exons and introns can be estimated using the scale at the bottom of the figure. **c.** Schematic representation of the conserved motifs in tomato MIOX proteins using MEME. Each color represents a specific motif, and each motif is represented as a numbered box. The length of the box does not correspond to the length of the motif

MIOX protein sequences of tomato and other species were aligned, and a phylogenetic tree was constructed. Multiple sequence alignment and phylogenetic analysis revealed close linkages of *S. lycopersicum* with other species MIOX proteins. Among all, *MIOX1* and *MIOX4* proteins shared the highest levels (70% identical at the amino acid level) of similarity (Fig. 1S).

### Gene structure, chromosomal locations, conserved motifs and expression profiles of MIOX genes

Tomato MIOX protein sequences were aligned (Fig. S2). The phylogenetic tree was constructed using MEGA5.0 with 1000 bootstrap replicates, which revealed that 5 MIOX were clustered into two groups. *MIOX1* and *MIOX5* were placed in one group, and *MIOX2* and *MIOX4* placed in another group together with *MIOX3* (Fig. 1a). The MIOX genes structure was determined using the GSDS database. The analysis revealed that all MIOX genes contain intron regions except *MIOX5*. The number of introns varies among MIOX genes, ranging from 1 to 10. *MIOX1* contains a maximum of 10 intron regions, followed by *MIOX4*, which contains eight intronic regions (Fig. 1b), while *MIOX3* contains seven intronic regions. However, *MIOX2* contains only one intronic region.

The conserved motifs of MIOX genes were identified using the MEME database. Motifs 1–4 are MIOX motifs, and most of these motifs are present in all MIOX genes, indicating that these MIOX proteins contain MIOX functional domains (Fig. 1c). Although motifs 5–7 were undetected in any known functional domains according to searches using Pfam and NCBI databases. Moreover, motifs 4 was absent in only the *MIOX5* gene, but all other MIOX motifs are present in *MIOX5* as well. The occurrence of a similar type of conserved motifs potentially indicates functional similarity among MIOX family members.

MIOX protein sequences were used to retrieve the chromosomal locations of genes in the tomato genome. The analysis revealed that MIOX genes are distinctly spread among the tomato chromosomes. Chromosome 6, 10, and 11 each contain one MIOX gene, while chromosome 12 contains two MIOX genes (Fig. S3).

### MIOX gene expression pattern in different organs

**Fig. 2:**
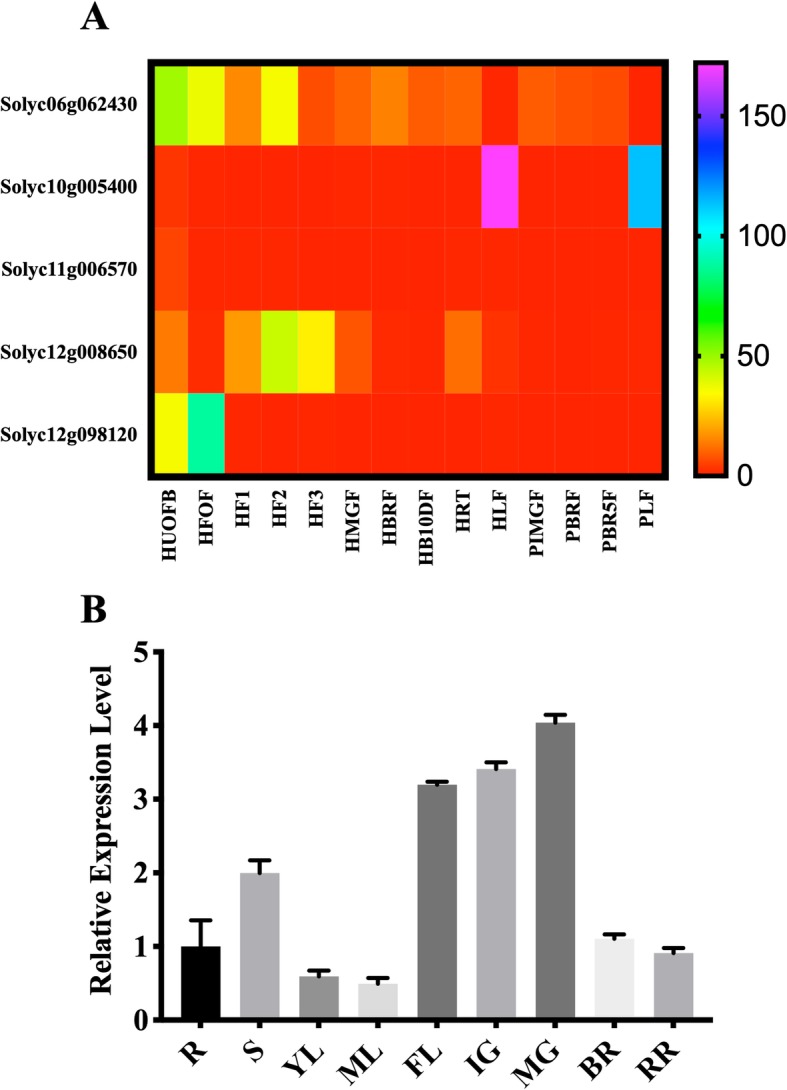
Expression patterns of the *MIOX* genes in tomato Heinz (cultivated type) and *pimpinellifolium* (wild-type). **a.** Organ-based expression profiles for *MIOX* genes of cultivar Heinz and *pimpinellifolium*. The color bar represents the log^2^ expression values, with Purple representing high expression levels and red representing low expression levels. The gene name is shown on the left side. Developmental stages used for expression profiling are indicated at the bottom of each column. Heinz unopened flower buds (HUOFB), Heinz fully opened flowers (HFOF), Heinz 1 cm fruits (HF1), Heinz 2 cm fruits (HF2), Heinz 3 cm fruits (HF3), Heinz mature green fruits (HMGF), Heinz breaker fruits (HBRF), Heinz breaker+ 10 fruits (HB10DF), Heinz roots (HRT), Heinz leaves (HLF), pimpinellifolium immature green fruits (PIMGF), pimpinellifolium breaker fruits (PBRF), pimpinellifolium breaker+ 5 fruits (PBR5F), pimpinellifolium leaves (PLF). **b.** Expression patterns of *MIOX4* in different Alisa Craig (AC) tomato tissues. R, root; S, stem; YL, young leave; ML, mature leave; FL, flower; IG, immature green; MG, mature green; BR, breaker; RR, red ripe

RNA-seq data was examined to reveal the expression pattern of different organs during the different developmental stages of tomato. The analysis showed that MIOX genes were expressed differently in all the examined tissues of *cultivar Heinz* (cultivated tomato) and *S. pimpinellifolium* (wild tomato) (Fig. 2a). *MIOX1* was highly expressed from flowering to the fruiting stage of cultivar Heinz, while *MIOX2* expression was observed only in leaves of both the cultivar (*Heinz*) and wild (*pimpinellifolium*) tomato. *MIOX3* exhibited very low expression, while *MIOX5* revealed a high expression pattern at the flowering stage of cultivar Heinz. *MIOX4* recorded high expression during flowering and early fruit stage with moderate expression in roots and leaves of cultivar Heinz. To further confirm the RNA-seq expression analysis, we performed an RT-qPCR to examine the expression pattern of *MIOX4* in different organs of tomato cultivar *S. lycopersicum* cv. Alisa Craig. The analysis revealed that *MIOX4* was highly expressed in the stem, flower, young, and green fruits. The expression level was, however, not high in roots, leaves, breaker, and red ripe fruits (Fig. 2b).

### MIOX overexpression altered the expression of AsA-related genes and AsA concentration in both leaves and fruits

**Fig. 3:**
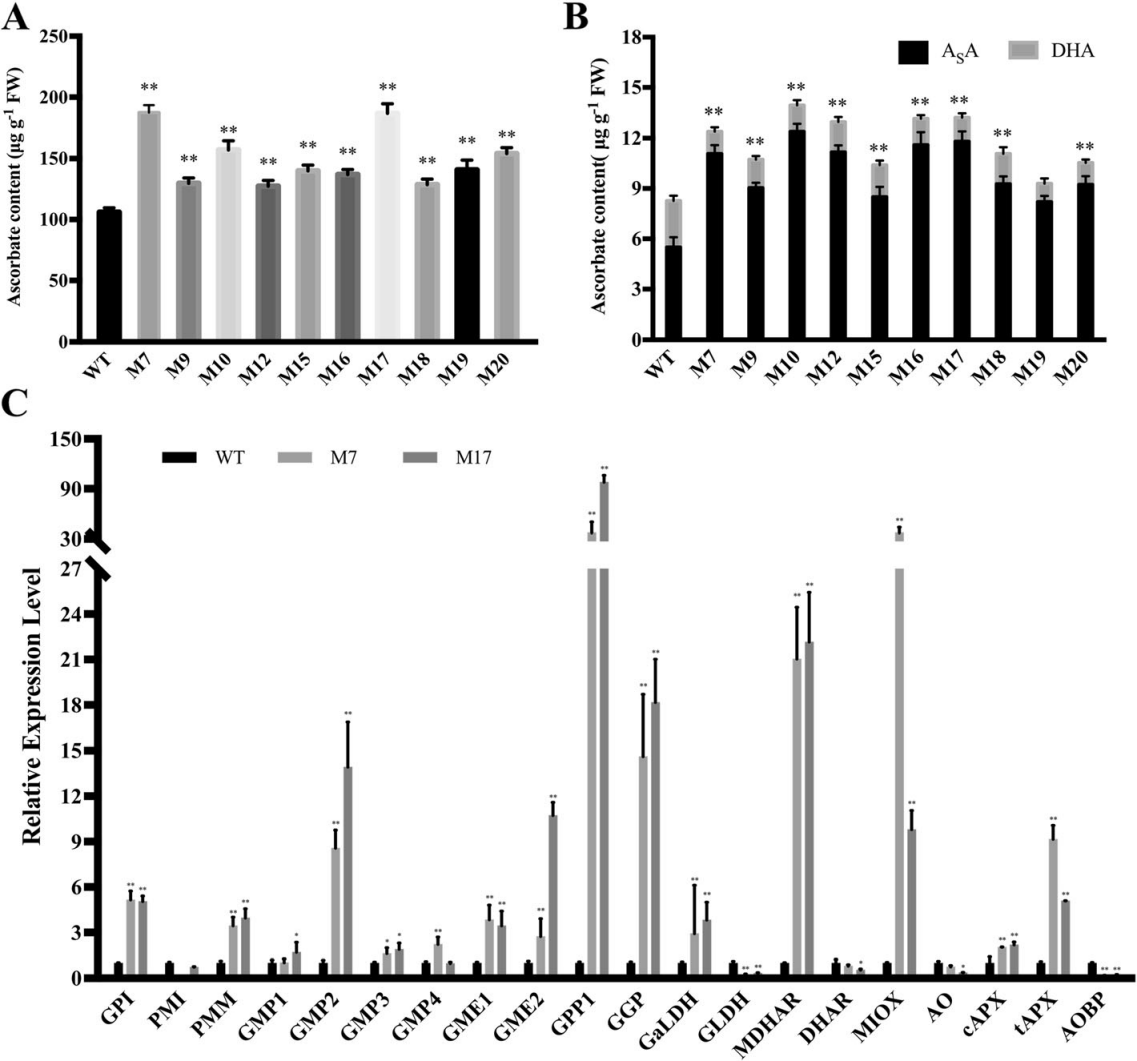
Ascorbate concentration in leaf and red ripe fruits of transgenic *MIOX4* tomato lines and relative expression of AsA biosynthesis, recycling-related genes. **a**. Ascorbate content in young leaf. **b**. Reduced and total ascorbate contents in red ripe fruits. **c**. Relative expression of AsA biosynthesis and recycling-related genes in leaf. *GPI* (glucose-phosphate isomerase, Solyc04g076090), *PMI* (phosphomannose isomerase, Solyc02g086090), *PMM* (phosphomannomutase, Solyc05g048760), *GMP1* (GDP-Man pyrophosphorylase, Solyc03g096730), *GMP2* (GDP-D-mannose pyrophosphorylase 2, Solyc06g051270), *GMP3* (GMP synthase, Solyc07g006090), GMP4 (GDP-Man 4 6-dehydratase, Solyc03g118270), *GME1* (GDP-Man-3′,5′-epimerase 1, Solyc01g097340), *GME2* (GDP-Man-3′,5′- epimerase 2, Solyc04g077020), *GPP1* (L-Gal 1-phosphate phosphatase 1, Solyc04g014800), *GGP* (GDP-L-Gal phosphorylase/L-Gal guanylyltransferase, Solyc06g073320), *GalDH* (L-Gal dehydrogenase, Solyc01g106450), *GLDH* (L-GalL dehydrogenase, Solyc10g079470), *MDHAR* (monodehydroascorbate reductase, Solyc09g009390), *DHAR1* (dehydroascorbate reductase, Solyc05g054760), *MIOX* (*myo*-inositol oxygenase, Solyc12g008650), AO (ascorbate oxidase, Solyc04g054690), cAPX (Cytosolic ascorbate peroxidase, Solyc06g005160), tAPX (thylakoid ascorbate peroxidase, Solyc11g018550), AOBP (ascorbate oxidase promoter-binding protein, Solyc02g088070). Three replicate experiments were performed. Error bars represent standard error, means ± SE. FW, fresh weight. The asterisks represent significant differences from wild-type (Alisa Craig), as indicated by the *t*-test (*P < 0.05; **P < 0.01)

MIOX4 sequence was isolated according to *S. lycopersicum* full-length cDNA and transformed in tomato Alisa Craig to generate the transgenic lines as described in methods. The AsA content and expression of AsA-related genes were examined in the wild-type and *MIOX4* overexpression transgenic lines (Fig. 3). The AsA level in leaves of MIOX overexpression lines showed a significant increase compared with the wild-type, and maximum AsA was observed in MIOX-M7 and MIOX-M17 transgenic lines (Fig. 3a). The substantial amount of ascorbate in MIOX transgenic lines signified that the biosynthesis of AsA might be subjected to feedback inhibition in tomato leaves and thus, limiting the increase of AsA up to a certain level. The total AsA and reduced AsA content in fruits of MIOX overexpression lines increased significantly compared with the wild-type (Fig. 3b); whereas, the overall increase in fruit ascorbate content was much lower than in leaves (Fig. 3a-b).

In addition to the increased expression of the *MIOX4* in the overexpression lines, the transcript level of most AsA biosynthesis and recycling genes showed a slight significant increase in fruits of two transgenic lines (Fig. 3c). In fruits, except *PMI, GLDH, DHAR, AO, Capx*, and *AOBP*, the other AsA-related structural genes such as *GMP2, GME2, GPP1, GGP, MDHAR, MIOX,* and *tAPX* showed a significant increase in *MIOX4* overexpression lines. These findings indicate that *MIOX4* transgenic lines contribute to the biosynthesis of AsA in tomato leaves and fruits in the _D_-Man/_L_-Gal pathway. This AsA biosynthesis could be subjected to feedback inhibition. Overexpression of the *MIOX4* gene increases the AsA content in transgenic lines but down-regulates the expression of some structural genes by feedback inhibition.

### Total AsA content in leaves and fruits of MIOX overexpression lines altered by synthetic precursor inositol (MI)

Previously reported results indicated that; an increase of AsA content in *MIOX4* transgenic lines is associated with the AsA biosynthesis pathway in leaves and fruits. Therefore, inositol (MI), a substance representing the AsA biosynthesis pathway was chosen to do the feeding experiment, H_2_O was used as a control. In leaves, MI feeding increased the AsA content in both MIOX transgenic lines and Alisa Craig. This increase was significant in both MIOX transgenic lines. On the contrary, the AsA content recorded no difference among Alisa Craig (AC) and transgenic lines in the mature green fruit stage. MI plays a particular role in AsA biosynthesis, suggesting that myo-inositol pathway was the main contributor to AsA accumulation in tomato leaves.

**Fig. 4:**
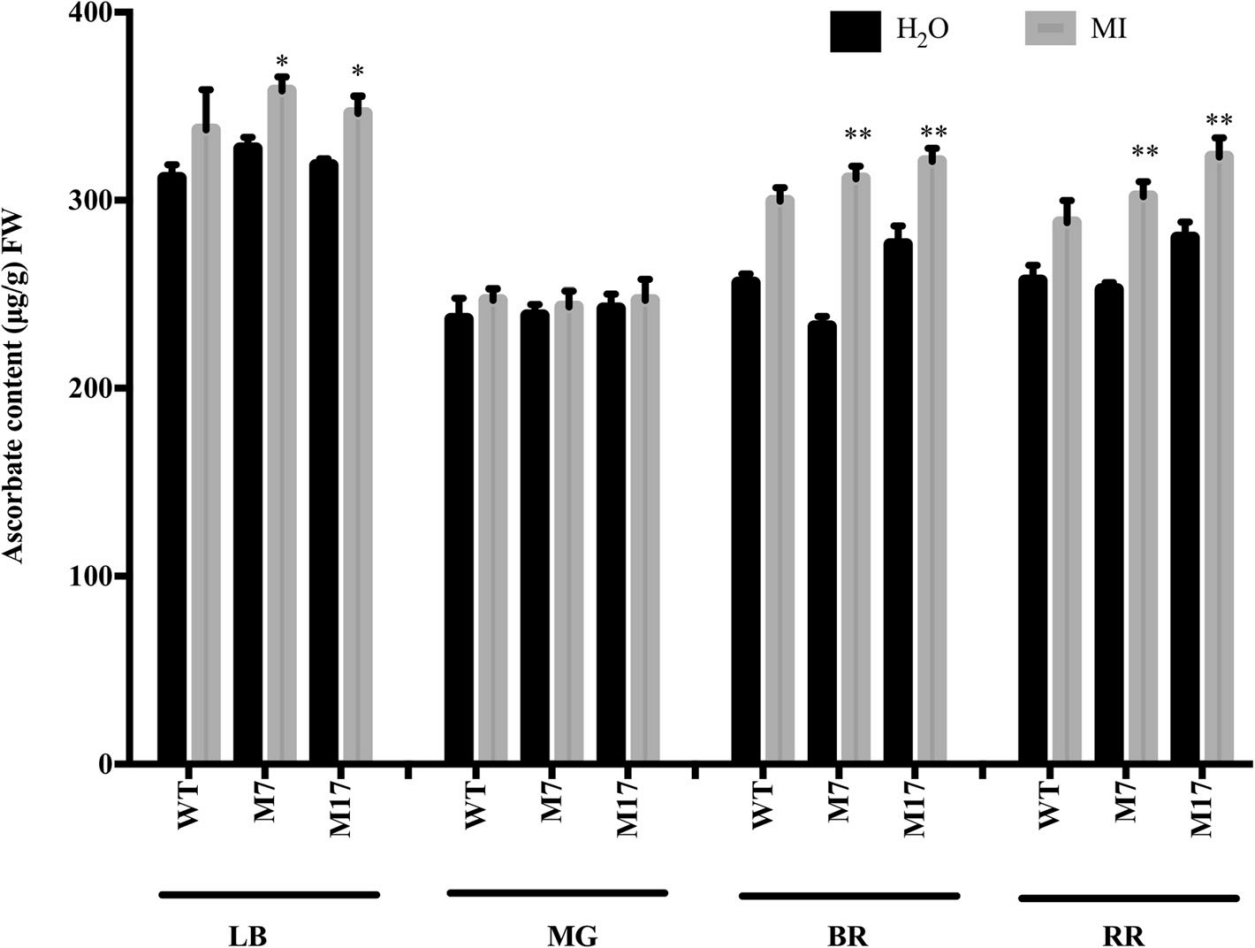
Total AsA content in leaves and fruit of *MIOX4* over-expression lines altered by Synthetic Precursor Inositol (MI). The leaves (LB) and fruit at mature green (MG), breaker (BR), and red ripe (RR) were fed with H_2_O and inositol (MI) and incubated under light for 24 h. Three replicates were performed; and data presented as means ± SE. FW, fresh weight. The asterisks represent significant differences from wild-type (Alisa Craig), as indicated by the t-test (*P < 0.05; **P < 0.01)

Meanwhile, MI feeding increased the AsA content in breaker and red ripe fruits of transgenic lines. However, the AsA content in the fruit of AC showed no change after feeding with MI (Fig. 4). These findings suggest that myo-inositol pathway contributes to AsA biosynthesis in tomato breaker and red ripe fruits in transgenic lines. MIOX overexpression may have improved the AsA biosynthesis pathway in which the myo-inositol pathway plays a role in leaves and fruits and consequently alters AsA content. Therefore, the increase in the AsA content of transgenic lines could be ascribed to the MIOX overexpression gene, which might have increased the overall MIOX activity.

### The light-dependent fluctuation of AsA content in wild-type and MIOX lines

**Fig. 5:**
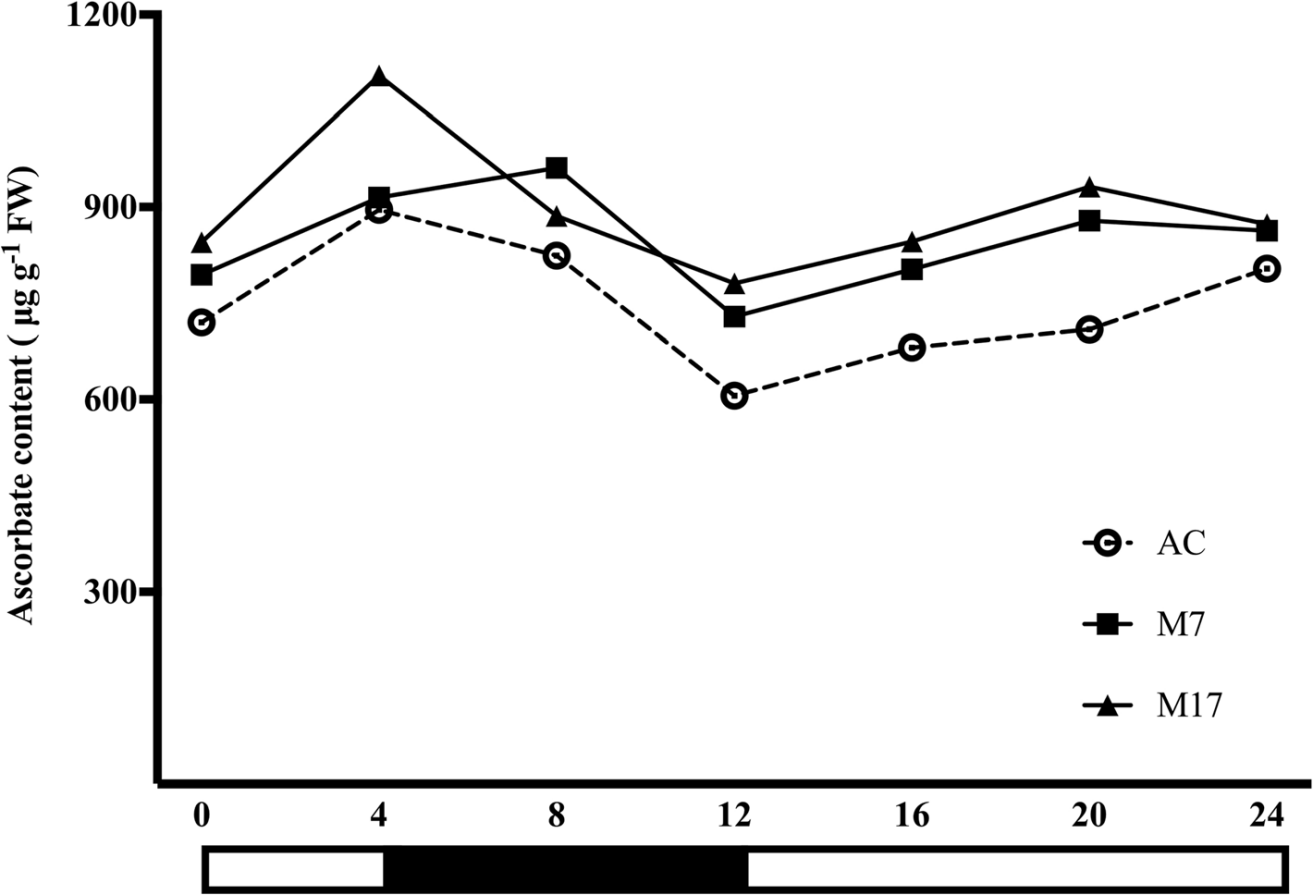
Dynamic change of ascorbate accumulation in response to light in leaves of Alisa Craig and *MIOX4* transgenic lines. Plants were grown in the greenhouse, and fully expanded leaves were harvested every 4 h, total ascorbate content was measured for 24 h. Three replicates were performed. Error bars represent standard error, means ± SE. FW, fresh weight

To further investigate the effect of MIOX transgenic lines on light-dependent ascorbate metabolism, we determined AsA content in the leaves under different light conditions for 24 h. Dynamic changes as a model of “up-down-up”, were observed in total AsA content of both wild-type and transgenic lines under the different light conditions for 24 h. However, AsA content in transgenic lines was significantly higher compared with the wild-type (Fig. 5). These results indicate photoperiod-specific changes in AsA content, affirming that light significantly contributes to AsA accumulation in tomato. We observed that AsA content increased in leaves under light condition up to a certain level but declined in extended illumination. Generally, MIOX overexpression improved the light-induced AsA accumulation in tomato leaves.

### MIOX overexpression increases AsA transport capacity in transgenic lines

**Fig. 6:**
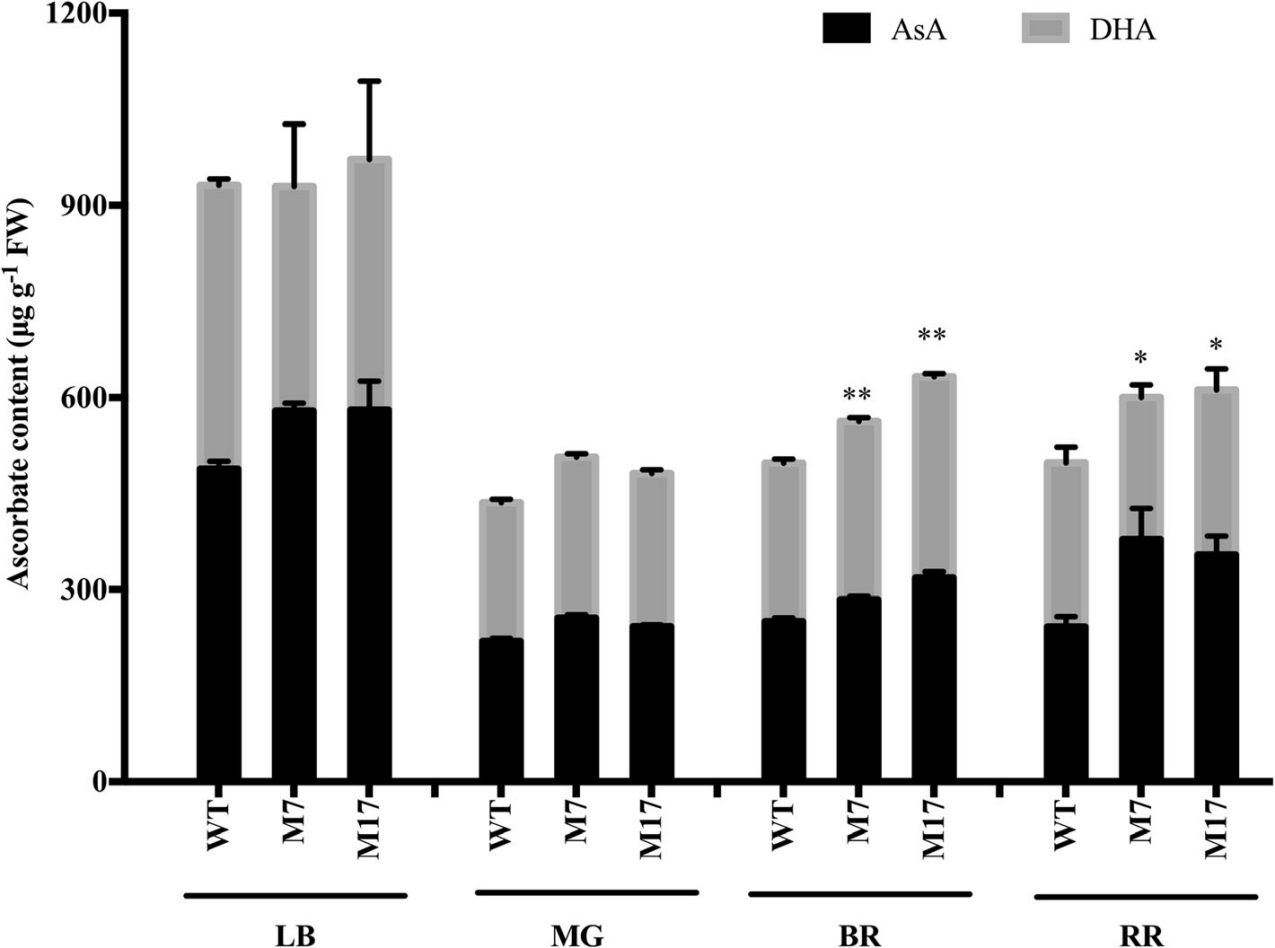
Ascorbate concentration in leaves and fruit petioles and their exudates of wild-type and *MIOX4* tomato lines. AsA levels in leaves (LB) and fruits at mature green (MG), breaker (BR), and red ripe (RR) of wild-type and MIOX lines. Three replicates were performed. Error bars represent standard error, means ± SE. FW, fresh weight. The asterisks represent significant differences from wild-type (AC), as indicated by the *t*-test (*P < 0.05; **P < 0.01)

The MIOX transgenic lines capability to affect AsA transport was measured as it has been reported earlier that AsA synthesis both in leaves and fruits are mostly transported from leaves to fruits [16]. Leaf petioles and fruit exudates of wild-type and MIOX transgenic lines were used to measure the AsA content. The analysis revealed that AsA content has no significant difference in leaf petioles and mature green fruit exudates of wild-type and MIOX transgenic lines. Interestingly, breaker and red ripe fruit exudates showed a significant increase of AsA content in transgenic lines compared with wild-type (Fig. 6), consistent with the AsA content in fruits. The findings indicate that the AsA content significantly increased in fruit secretion and leaf petioles of MIOX transgenic lines compared with wild-type.

### MIOX overexpression improved tolerance to oxidative stress in tomato

**Fig. 7:**
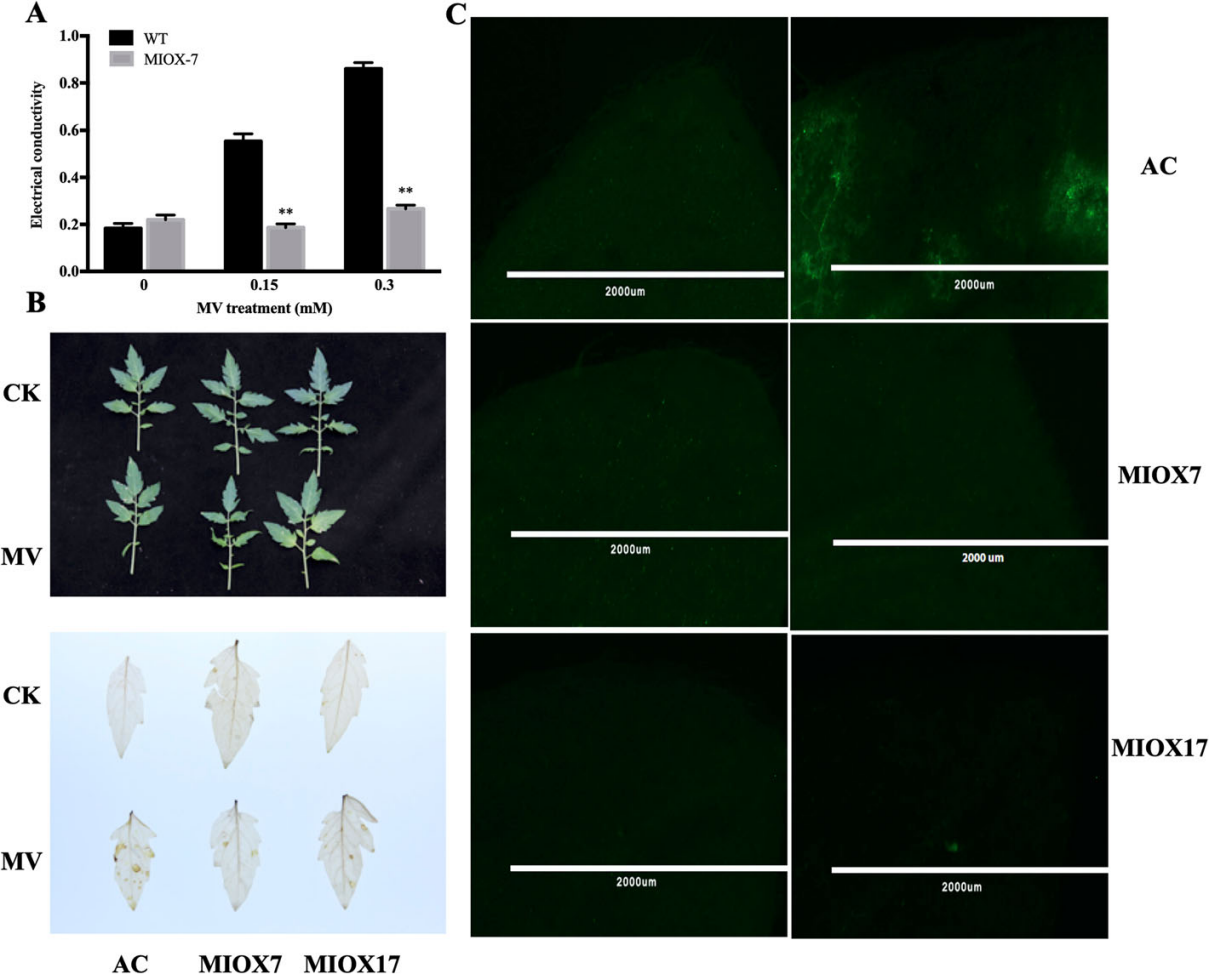
The oxidative stress tolerance in Alisa Craig and *MIOX4* tomato lines. **a.** Electric conductivity (EC) in leaves treated with methyl viologen (MV) or water (CK) was measured on day 3 and 7 post-treatment, respectively. Three replicates were performed; and data presented as means ± SE. The asterisks represent significant differences from the control (CK), as indicated by the *t*-test (*P < 0.05; **P < 0.01). **b.** Detection of H_2_O_2_ accumulation in the leaves of Alisa Craig and MIOX lines by DAB staining in leaves from the two-month-old plants of Alisa Craig and *MIOX4* lines. **c.** H2DCFDA fluorescence was performed to reveal the accumulation of H_2_O_2_ in leaves from the two-month-old plants of Alisa Craig and *MIOX4* lines

Ascorbic acid (AsA) is an antioxidant involved in the removal of reactive oxygen species (ROS) and protects plants from oxidative damage. Mostly, increased AsA content help plants to resist oxidative stress. To examine whether AsA biosynthesis-related MIOX transgenic lines in tomato can increase oxidative stress tolerance, 4-weeks-old transgenic lines and wild-type were exposed to oxidative stress by spraying different concentrations (0.15 and 0.30 mM) of methyl viologen (MV), which can imitate the oxidation environment, for 48 h. The electrical conductivity (EC) showed the damage caused by MV. After MV treatments, EC in the wild-type plants showed a significant increase, whereas no significant changes were found in MIOX transgenic lines. After MV treatments, EC was significantly reduced in transgenic lines (66% in 0.15 mM MV and 69% in 0.30 mM MV) compared with wild-type plants (Fig. 7a).

Furthermore, DAB staining and H_2_DCFDA fluorescence were observed to determine the accumulation of H_2_O_2_ in leaves of wild-type and MIOX transgenic lines. DAB staining analysis showed no significant difference between Alisa Craig and MIOX transgenic lines after treatment with H_2_O. Significant brown spots were observed on the leaves of Alisa Craig compared with the leaves of MIOX transgenic lines after MV treatments (Fig. 7b). The H_2_DCFDA fluorescence results showed similar results with no significant difference between Alisa Craig and MIOX transgenic lines treated with H_2_O. At the same time, Alisa Craig generated brighter fluorescence than MIOX transgenic lines after MV treatments (Fig. 7c). In general, it seems that AsA related MIOX transgenic lines increased oxidative stress tolerance in tomato.

### MIOX overexpression altered the water holding capacity of transgenic lines

**Fig. 8:**
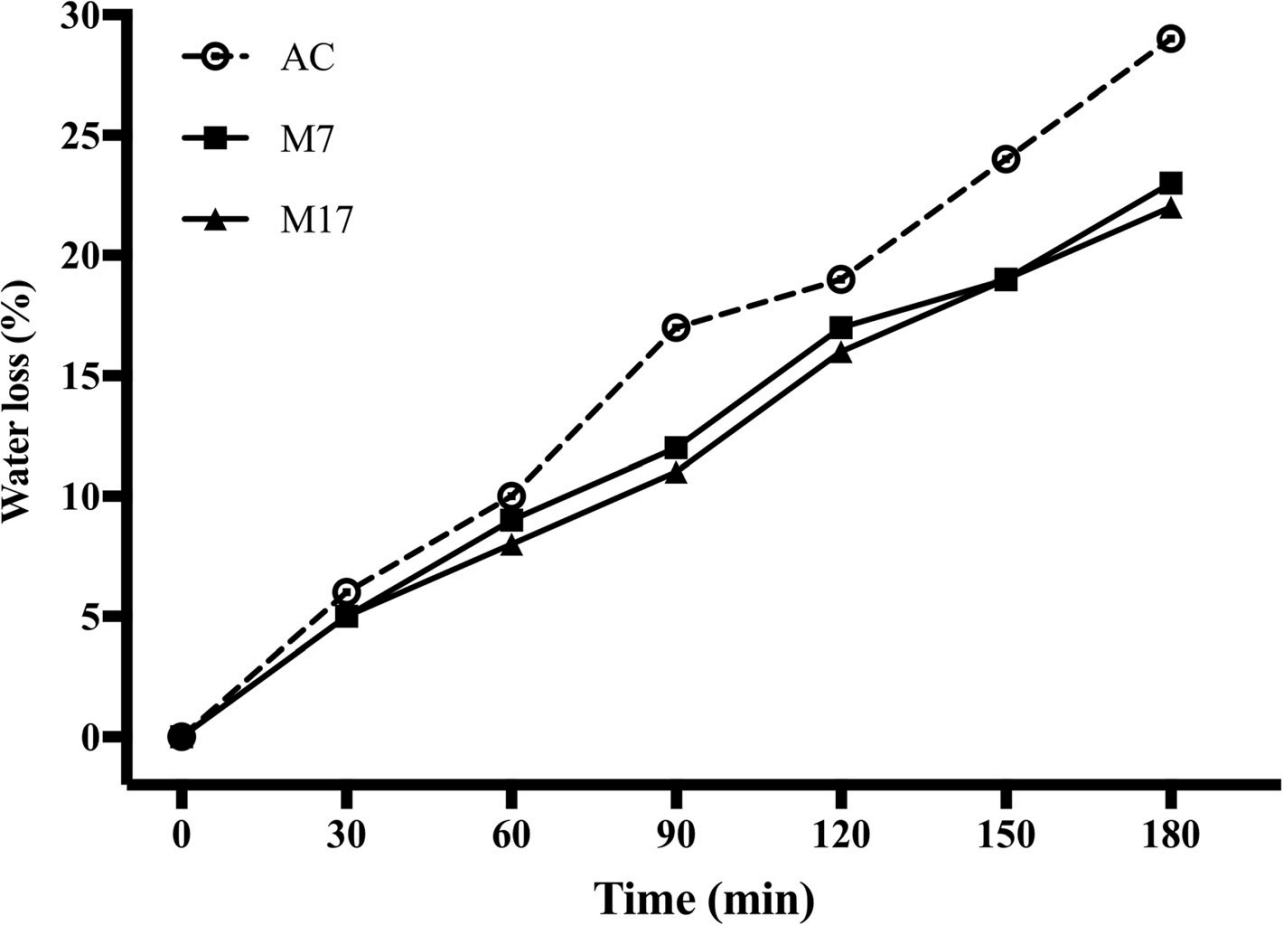
Change of water content in leaves of Alisa Craig and *MIOX4* transgenic lines. Water loss of the detached leaves from Alisa Craig and *MIOX4* transgenic lines. Water loss is represented as the percentage of initial fresh weight at each time point. Values are means ± SE of 25 leaves from five plants of each Alisa Craig or transgenic line. Asterisks indicate significant differences between transgenic lines and wild-type. Three replicates were performed

It has been well reported that AsA is one of the most abundant water-soluble antioxidant compounds for enhancing abiotic stress tolerance in plants [17]. To evaluate MIOX transgenic lines response to stress conditions, 4-weeks-old transgenic lines, and leaves of wild-type plants were exposed to dehydration stress. The analysis revealed that after the dehydration stress, wild-type plants showed higher water loss (26%) compared with transgenic lines (Fig. 8); thus, MIOX tomato transgenic lines exhibit increased desiccation stress tolerance.

### Morphological attributes affected by MIOX overexpression

**Fig. 9:**
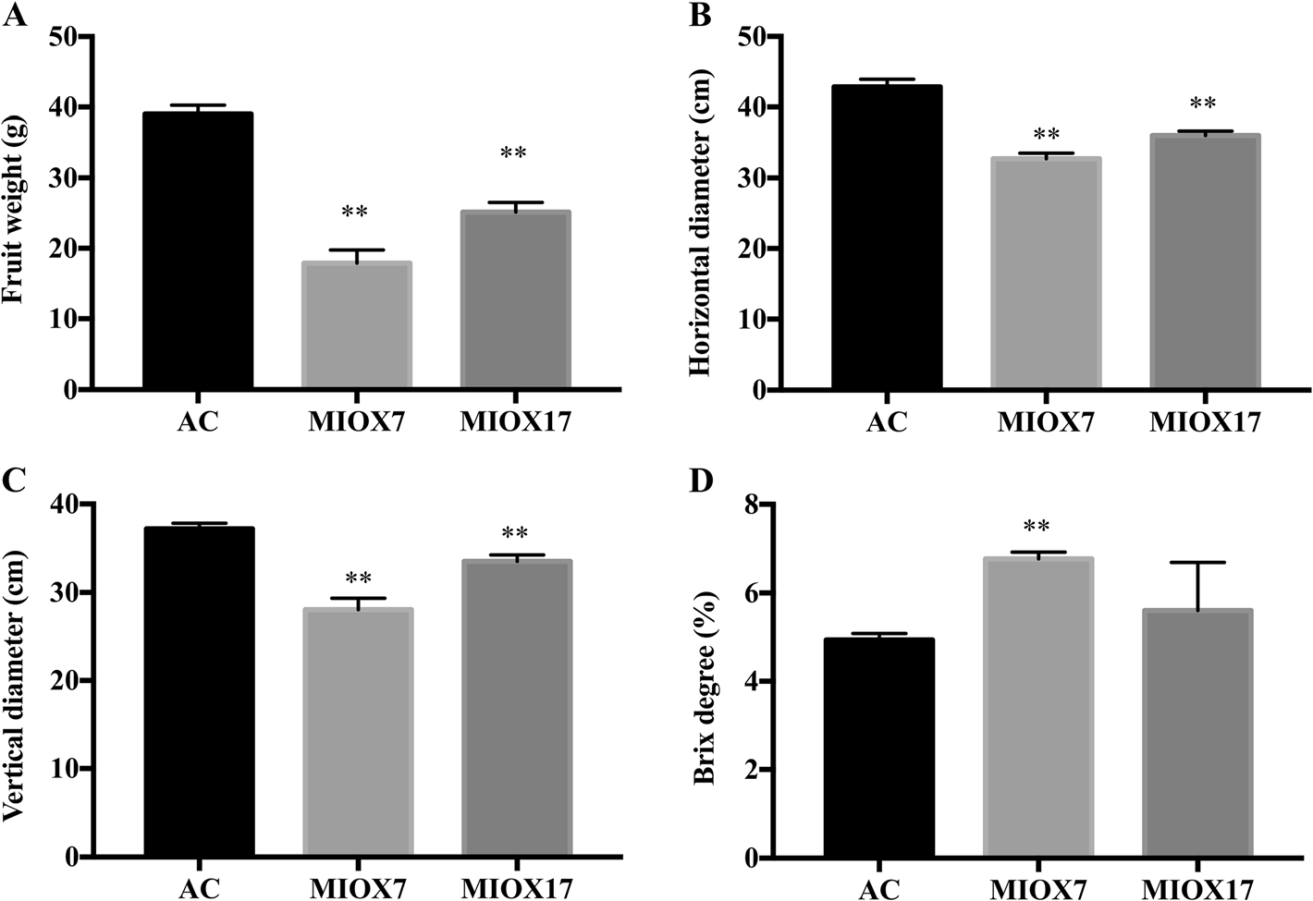
The fruit weight (**a**), horizontal diameter (**b**), vertical diameter (**c**) and Brix (**d**) in Alisa Craig and *MIOX4* lines. Three replicates were performed. Error bars represent standard error, means ± SE. The asterisks represent significant differences from wild-type (*pimpinellifolium*), as indicated by the *t*-test (*P < 0.05; **P < 0.01)

Remarkably, other than improved AsA accumulation, MIOX transgenic lines showed a noticeable difference in fruit weight, size, shape, and Brix level compared with Alisa Craig (Fig. 9). The fruit weight, the horizontal and vertical diameter of MIOX transgenic lines was significantly reduced compared to Alisa Craig (Fig. 9a-c). The Brix level of MIOX transgenic line (M7) was significantly higher than Alisa Craig (Fig. 9d). These findings demonstrate that MIOX transgenic lines could significantly affect the overall appearance, quality, and Brix level of tomato fruits.

## Discussion

Ascorbic acid (AsA) is an important antioxidant in plants, which is crucial in the removal of reactive oxygen species (ROS). Humans take AsA in food as vitamin C, which has gained research prominence in AsA biosynthesis and regulatory mechanism in plants. Myo-inositol oxygenase (MIOX) is a key enzyme in plants AsA biosynthesis pathway [18]. Though, animals have a single MIOX gene, while plant houses gene families encoding the MIOX enzymes. For instance, *Populus trichocarpa* (poplar) contains three predicted MIOX genes [19], *Oryza sativa* (rice) contains two predicted MIOX genes [20], and *Arabidopsis thaliana* contains four MIOX genes, named according to their chromosomal locations (*MIOX1*: At1g14520; *MIOX2*: At2g19800; *MIOX4*: At4g26260; *MIOX5*: At5g56640) [8, 21].

In this study, we have reported five MIOX genes in tomato and named them in accordance with their chromosomal locations (*SlMIOX1*: Solyc06g062430.3; *SlMIOX2*: Solyc10g005400.3; *SlMIOX3*: Solyc11g006570.2; *SlMIOX4*: Solyc12g008650.2; *SlMIOX5*: Solyc12g098120.2) (Table 1). The tomato MIOX members are 66–80% identical compared to Arabidopsis four MIOX isoforms, which are 66–84% identical at the amino acid level [8]. Phylogenetic analysis of five tomato with other species MIOX proteins revealed that *MIOX1* and *MIOX4* are 70% identical at the amino acid level between tomato and Arabidopsis (Fig. 1S). The MIOX genes structure analysis revealed that Arabidopsis *MIOX* acquired more intronic regions during gene-duplication events compared to tomato MIOX genes. The exon/intron structure in Arabidopsis and tomato evidence multiple MIOX gene duplication events during the evolution of plants. However, MIOX genes in tomato have retained their MIOX functional domains (Fig. 1c) during these evolutionary events, as in Arabidopsis MIOX genes. MIOX genes in tomato are distinctly spread among tomato chromosomes 6, 10, 11, and 12. whereas, Arabidopsis MIOX genes were present on chromosomes 1, 2, 4, and 5 [8]. These variations in intron/exon organization and distribution of MIOX genes on chromosomes indicate the potential dissimilarities in the function of the tomato MIOX genes compared with Arabidopsis.

Presently, there are various studies on MIOX role in plants with conflicting findings reported as earlier research has suggested two different opinions on the MIOX role in the AsA biosynthesis pathway. In the first opinion, overexpression of *MIOX4* increased the ascorbate content in leaves by two to three times, suggesting that overexpression of MIOX can improve AsA content in Arabidopsis [15]. On the other hand, overexpression of MIOX increases MIOX enzyme activity by more than 30-fold with unaltered ascorbic acid content in Arabidopsis [13]. More recently, another research has demonstrated that; the Myo-inositol pathway does not contribute to ascorbic acid synthesis in Arabidopsis [2]. These two results are contradictory. However, the results of this study demonstrated that overexpression of *SlMIOX4* in tomato significantly increases the enzyme activity of MIOX and also increases the AsA content in tomato leaves and fruits (Fig. 3). Therefore, our findings are inconsistent with these viewpoints, as feeding MI to positive transgenic plants results in less accumulation of myo-inositol in transgenic lines than that of the control. The previous study also showed no difference in the AsA product of transgenic lines and the control. The amount of MIOX overexpression only contributed to an increase in enzyme activity [13]. The results of this study further highlight that feeding MI contributes to the amount of AsA produced in *MIOX4* overexpression transgenic lines. We observed a significantly higher level of AsA in transgenic lines than that of the control plants (Fig. 6). This study contributes to understand the molecular mechanism of *MIOX4* transgenic lines for the increase of AsA content in tomato plants. Based on our findings, we assumed that the potential reason for the increase of AsA content in *MIOX4* transgenic lines could be multifactorial. In this study, we observed an up-regulated expression level for most of the AsA biosynthesis-related genes in the leaves of MIOX overexpression lines compared with control plants (Fig. 3).

Therefore, the stimulated AsA pathway could be one of the reasons for the increase of AsA content in *MIOX4* overexpression lines. Furthermore, our analysis showed an increase in the amplitude of fluctuation during the period of 24 h in *MIOX4* overexpression without completely altering the basic rule of the circadian rhythm (Fig. 5). This may be ascribed to the overexpression of *MIOX4* enhancing plant sensitivity to light and increased the photosynthetic substrate product, which is indispensable in the basic biosynthetic pathway of AsA [4], and thus, contributing to increased accumulation of AsA in plants. Light intensity is considered one of the critical environmental factors affecting AsA accumulation in plants [22]. Besides, the MI feeding experiment revealed the contribution of the myo-inositol pathway to AsA synthesis in the leaves of Alisa Craig and *MIOX4* overexpression tomato lines (Fig. 4). Moreover, in the *MIOX4* overexpression lines, the biosynthesis genes also increased the transport capacity of AsA. Overall, our findings demonstrate that *MIOX4* overexpression enhances the synthesis and transport capacity of AsA in tomato.

MIOX is a vital mono-oxygenase involved in the conversion of *myo*-inositol into d-glucuronic acid (d-GlcUA) and plays a significant role in AsA biosynthesis in plants. An increased amount of AsA can enhance plant resistance to oxidative damages [23, 24]. In this study, we evaluated the *MIOX4* overexpression lines against abiotic stress (Fig. 7). The results showed that; tolerance to oxidative stress was enhanced in *MIOX4* overexpression lines, consistent with previous reports [14, 23, 25, 26]. Besides the enhanced antioxidant capacity, MIOX overexpression lines showed altered fruit morphology, such as fruit size, fruit shape, and soluble solids (Fig. 9). Previous studies have shown that MIOX contains antioxidant properties, but also acts as a co-factor for numerous enzymes through evolving AsA and further contributes to the regulation of cell division and expansion [8, 13, 15]. It has been well-reported that *AtMIOX4* overexpression leads to improved accumulation of ascorbic acid [1, 15, 27]. Additionally, the ascorbate pathway is involved in the primary metabolism pathway and subsequently influences organic acid and sugar metabolism in tomato [28].

## Conclusions

In this study, *MIOX4* overexpression lines showed an enhanced AsA accumulation and improved tolerance to abiotic stress conditions. *MIOX4* overexpression lines exhibited improved light response, stress tolerance, and AsA transport capacity. Furthermore, MI feeding experiment showed myo-inositol biosynthesis pathway involvement in AsA accumulation in tomato. These findings are consistent with the previous reports, which indicate the significance of MIOX genes. MIOX is a potential gene family for the development of morphologically improved and nutritionally rich crops. The results of this study provide a base for genetic improvements in fruit quality and/or stress tolerance in tomato. Hence, future studies may focus on determining the in-depth functional mechanism of MIOX genes for developing commercially desired attributes of tomato.

## Methods

### Plant materials

Seeds of tomato cultivar (*S. lycopersicum* cv. Alisa Craig) were obtained from our lab. Plants were grown under controlled conditions of 24–28 °C temperature, natural light, and 70–80% relative humidity in a greenhouse for 5 weeks. Plant root, stem, leaves, flower, and fruit at different stages were collected. Three biological replicates of each line were analyzed, and each biological replicate contained three individual samples (leaves, fruits, and petioles of leaf and fruit) of the same developmental stage. All the samples were quickly frozen in liquid nitrogen to store at − 80 °C until further analysis.

### Identification of tomato MIOX genes

To identify myo-inositol oxygenase (MIOX) proteins in tomato, Arabidopsis (4) MIOX protein sequences were used as queries for homologous sequences with BLASTP (with an E cut off value 1e-5) search in the tomato genome database (Solanaceae Genomics Network, http://solgenomics.net/). Homology search was performed with keywords like ‘myo-inositol oxygenase’ and ‘MIOX’ in the tomato database. Domain analysis was performed using the Hidden Markov Model of Simple Modular Architecture Research Tool SMART (http://smart.embl-heidelberg.de/), InterProScan (http://www.ebi.ac.uk/Tools/pfa/iprscan5/), Pfam (http://pfam.xfam.org) and NCBI Conserved Domain Database (http://www.ncbi.nlm.nih.gov/cdd) programs. Proteins containing MIOX motifs or those present in MIOX protein family holding domains like IPR007828, and PF05153 were retrieved. The amino acids, nucleotide sequences, and other relevant information about each MIOX gene were retrieved from SGN (http://solgenomics.net/). The physio-chemical data comprising the theoretical isoelectric points (pi), molecular weight, and sequence length were retrieved from the ExpasyProtParam server (http://web.expasy.org/protparam/).

### Sequence alignment and phylogenetic analysis

The retrieved MIOX protein sequences were aligned with MUSCLE by using default settings for tomato, Arabidopsis, and other plant species. The phylogenetic tree construction was performed by PhyML and visualized through TreeDyn [29].

### Gene structure, chromosomal locations, and conserved motifs

Gene structure display server (GSDS) program with default settings was used to determine the exon and intron structures of the MIOX genes (http://gsds.cbi.pku.edu.cn/). Subcellular localization was predicted using the Wolf PSORT (http://www.genscript.com/psort/wolf_psort.html) program. The MIOX genes sequence was used to retrieve their chromosomal locations in the tomato genome database (Solanaceae Genomics Network, http://solgenomics.net/). The chromosomal locations of MIOX genes were visualized using the MAPCHART program. Conserved MIOX motifs were identified using the MEME suite (http://alternate.meme-suite.org/tools/meme) with (1) a maximum of 15 motifs and (2) an optimum motif width between 6 and 50 aa.

### Expression profiles of the MIOX genes

The MIOX genes expression profile was determined using the RNA-seq data retrieved through the tomato functional genomics database (http://ted.bti.cornell.edu/cgi-bin/TFGD/digital/home.cgi). This data contains various tissue expression in tomato, including unopened flower buds (HUOFB), fully opened flowers (HFOF), 1 cm fruits (HF1), 2 cm fruits (HF2), 3 cm fruits (HF3), mature green fruits (HMGF), breaker fruits (HBRF), breaker+ 10 fruits (HB10DF), roots (HRT), leaves (HLF) for cultivar Heinz 1706, and immature green fruits (PIMGF), breaker fruits (PBRF), breaker+ 5 fruits (PBR5F) and leaves (PLF), for the wild species, *S. pimpinellifolium* (LA1589). For gene expression of different organs of tomato at the various developmental stages, the normalized PKRM values were used to generate heat map using the GraphPad Prism software.

### *MIOX4* cloning and overexpression gene construct

Total RNA was isolated using TriZol reagent (Invitrogen, USA) from different tissues of tomato. The first-strand cDNA was obtained using M-MuLV Reverse transcriptase (Thermo Scientific) according to the manufacturer’s instructions. The MIOX specific primer (Table S1) were designed according to *S. lycopersicum* full-length cDNA (GenBank accession no. NM_001247664.1). The open reading frame (ORF) of MIOX was amplified using the cDNA of *S. lycopersicum*. Amplified PCR product was inserted into a pBI121 cloning vector, and subsequently, the recombinant clones were sequenced to identify the correct sequence. The SoftBerry tool (http://linux1.softberry.com/berry.phtml) was used for gene prediction, and sequence alignment was done by ClustalW2 (http://www.ebi.ac.uk/Tools/msa/clustalw2/).

The pBI121 cloning vector containing the correct and complete ORF was double-digested using SalI and SacI. The recovered fragment was ligated into the plant binary vector pMV digested/cleaved with XhoI and SacI. The MIOX gene fragment was used to replace the GUS gene of the expression vector plasmid pMV, which was downstream of the CaMV35s promoter and driven by the cauliflower mosaic virus (CaMV) 35S promoter. The construct was transformed into *Agrobacterium* strain LBA4404 by electroporation and used for transformation on tomato Alisa Craig (AC) via the standard leaf disk protocol. Primary tomato plants (T_0_) were selfed, and T1 seeds were collected. These transformed plants were confirmed by PCR using CaMV 35S promoter-specific forward and MIOX reverse primer (Table S1). Segregation analysis was performed to select homozygous lines as described previously [30]. For expression analysis of MIOX transgenic lines, quantitative real-time PCR (qRT-PCR) was performed. For further study, two independent homozygous lines (M7 and M17) of MIOX with higher expression levels were selected. The primers used in this study are listed in Table S1.

### Determination of AsA content in transgenic tomato plants

Approximately 200 mg of powdered leaf sample and 400 mg of powdered fruit sample were used for total AsA and reduced AsA determination. Ascorbic acid content was determined using InfiniteM200 (Tecan, http://www.tecan.com/) at 550 nm wavelength according to a previously described method [31]. Tomato fruits or leaves sample were lyophilized in liquid nitrogen and homogenized in 1 mL of ice-cold 6% trichloroacetic acid (TCA). After a 15-min extraction on ice, for total AsA levels, 20 μL of the supernatant was transferred to wells of a microtiter plate containing 20 μL of 5 mM dithiothreitol (DTT). After 20 min of incubation at 37 °C, 10 μL of *N-*ethylmaleimide (NEM) was added and followed by incubation for 1 min at room temperature. Eight μL of the color reagent was then added to the mixture and incubated for 1 h at 37 °C. Total AsA content and reduced AsA were determined by the same volume of 0.4 mol potassium phosphate buffer (pH 7.4) while the rest of the procedure was as for the total AsA assay. All used reagents were prepared, as previously described [31]. Two independent homozygous lines (M7 and M17) of MIOX with higher AsA levels were selected for further analyses.

### AsA content in leaves and fruits of transgenic plants

To determine the difference between leaves and different fruit developmental stages of AsA production, 4-week-old seedlings of transgenic T1 generation and control AC plants grown under control conditions were used. For AsA measurement, control AC and two transgenic lines (M7 and M17) with three replicates were used. The samples were collected from leaves, mature green, breaker, and red ripe fruit stage, and AsA content measured.

### Expression analysis of AsA-related genes in MIOX transgenic lines

To determine the expression pattern of AsA metabolism-related genes, Quantitative Real-Time PCR (qRT-PCR) was performed using the MIOX over-expression transgenic lines and wild-type plants grown under control conditions. Approximately, 100 mg powdered leaves and 200 mg powdered fruits were used for RNA extraction. Complementary DNAs were synthesized from total RNA using the HiScript® II Reverse Transcriptase (Vazyme, USA) following the manufacturer’s protocol. The expression levels of AsA metabolism-related genes were evaluated using LightCycler 480 instrument (Roche Diagnostics, Basel, Switzerland) and SYBR Green I Master Kit (Roche, http://www.roche.com/) according to the supplier’s instructions. The thermo-cycling conditions for real-time PCR were: 95 °C for 30 s; 40 cycles of 95 °C for 5 s, and 60 °C for 25 s. All the experiments were performed in triplicates. Tomato actin gene (GenBank ID: BT013524) was used as an internal control. Real-time PCR data were analyzed using the 2^-ΔΔCT^ method. The primers were designed by Primer3 (http://frodo.wi.mit.edu/primer3) and listed in Table S1.

### Synthetic precursor MI feeding of leaves and fruits

Four-week-old MIOX transgenic lines (M7 and M17) and wild-type (Ailsa Craig, AC) plants were used for synthetic precursor feeding of leaves by the leaf-disc method. The cut leaves by puncher were placed in a Petri dish containing 20 ml of 5 mM MI (Inositol), and leaves were incubated in water as a control. The Petri dishes were placed in a growth chamber under a 16 h/8 h light/dark cycle at 25 °C for 24 h. After incubation, leaves were washed three times with distilled water, gently dried, and then frozen in liquid nitrogen and stored at − 80 °C. For each line, three biological replicates were analyzed. For mature green, breaker, and red ripe fruit stage feeding analysis, the fruit stalk instead of leaf-disc was dipped in 20 ml of 5 mM MI (Inositol). Fruits incubated in water were used as a control. At the end of the incubation period, the stalk was detached from the fruit, and the fruit was washed with distilled water twice and mopped gently, and the pericarp was frozen in liquid nitrogen and stored at − 80 °C for further analysis.

### Water loss assay

In vitro leaf dehydration test was performed with slight modifications according to the previously described method [32]. Twelve leaves were collected from four plants of wild-type and MIOX transgenic lines (M7 and M17) and divided into three groups. The initial weight of each group of leaves was calculated and incubated at room temperature. These leaves were weighed periodically at an interval of 30 min up to 3 h. Water loss was estimated as the percentage of the control.

### Oxidative stress and electrical conductivity measurement

To determine the performance of MIOX transgenic line under oxidative stress, leaves of AC and transgenic lines were thoroughly sprayed with 0.15 mM and 0.30 mM methyl violet (MV) (MV dissolved in water with 0.1% Tween-20) or water with 0.1% Tween-20 (control) once a day for 2 days. After treatment for 1 week, leaves were collected and lyophilized in liquid nitrogen for the determination of electrical conductivity (EC). Meanwhile, the treated fresh leaves were used for DAB staining and H_2_DCFDA fluorescence.

The electric conductivity was measured as described previously [33]. Fresh leaves were obtained from the same part of the seedlings, 0.5 g weighed, 20 ml of distilled water added, and completely submerged in the solution. Shake the solution on a shaker for 24 h, and EC was measured using an electrical conductivity meter at a constant temperature (20–25 °C). After the first measurement, the solution was placed in a boiling water bath at 100 °C for 15mins to kill plant tissue and naturally cooled to measure the EC of this solution with a conductivity meter at a constant temperature (20–25 °C). The EC measured before and after boiling was used to measure the actual electrical conductivity of tomato plants.

MV treated leaf samples were stained with 3, 3′-diaminobenzidine (DAB) solution to detect the presence of hydrogen peroxide (H_2_O_2_), as previously described [34]. For H_2_DCFDA fluorescence analysis, MV treated leaf samples were immersed in 25 μM H_2_DCFDA solution for 15 mins in dark place and then washed three times with 20 mM phosphate buffer (pH 6). Finally, H_2_DCFDA fluorescence was detected and photographed using a non-eyepiece fluorescence microscope (NIKON ECLIPSE 80i, Japan).

### Morphological attributes

Seedlings grown in a growth chamber with control conditions were used to measure the morphological attributes. Fruit weight (g), fruit horizontal (cm), vertical diameter (cm), and soluble solutes (%) were measured using two transgenic lines (M7, M17) and wild-type AC plants.

### Data analysis

For all experiments, samples were biologically replicated at least three times, and results were represented as means with standard error. Raw data were collated using Microsoft excel (ver. 20,016), and statistical significance was calculated by Student’s *t*-test at the *P* < 0.05 and *P* < 0.01 levels using GraphPad Prism software.

## Supplementary information

**Additional file 1 Table S1.** List of primer sequences. **Fig. S1.** Phylogenetic analysis of 5 MIOX proteins from *S. lycopersicum* with other species MIOX proteins. **Fig. S2.** Alignment of *5* MIOX protein sequence from *S. lycopersicum*. Multiple sequence alignments were conducted using ClustalW2. **Fig. S3**. Chromosomal distribution of the *SlMIOX* genes. A total of 5 *SlMIOX* genes were mapped onto tomato chromosomes. The gene identity numbers are provided, and respective chromosome numbers are shown at the top.

## Data Availability

All data generated or analyzed during this study are included in this published article [and its supplementary information files].

## Abbreviations

AC: *S. lycopersicum* cv. Alisa Craig
AsA: Ascorbic acid
MIOX: myo-inositol oxygenase
MI: Myo-inositol
MIOP: MI oxidation pathway
MIPS: L-myo-inositol 1-phosphate synthase
IMPase: Inositol monophosphate phosphatase
PtdIns: Phosphatidylinositol
SGN: Sol Genomics Network
GSDS: Gene structure display server
cDNA: Complementary DNA
ORF: Open reading frame
CaMV: Cauliflower mosaic virus
qRT-PCR: Quantitative real-time PCR
TCA: Trichloroacetic acid
DTT: Dithiothreitol
NEM: *N-*ethylmaleimide
MV: Methyl violet
EC: Electrical conductivity
DAB: 3, 3′-diaminobenzidine
H_2_O_2_: Hydrogen peroxide
ROS: Reactive oxygen species
HUOFB: Cultivar Heinz 1706 unopened flower buds
HFOF: Cultivar Heinz 1706 fully opened flowers
HF1: Cultivar Heinz 1706 1 cm fruits
HF2: Cultivar Heinz 1706 2 cm fruits
HF3: Cultivar Heinz 1706 3 cm fruits
HMGF: Cultivar Heinz 1706 mature green fruits
HBRF: Cultivar Heinz 1706 breaker fruits
HB10DF: Cultivar Heinz 1706 breaker+ 10 fruits
HRT: Cultivar Heinz 1706 roots
HLF: Cultivar Heinz 1706 eaves
PIMGF: *S. pimpinellifolium* (LA1589) immature green fruits
PBRF: *S. pimpinellifolium* (LA1589) breaker fruits
PBR5F: *S. pimpinellifolium* (LA1589) breaker+ 5 fruits
PLF: *S. pimpinellifolium* (LA1589) leaves
