# Supplementary material for: Genome-wide analysis of Myo-inositol oxygenase gene family in tomato reveals their involvement in ascorbic acid accumulation
Source: BMC Genomics. 2020 Apr 6;21:284. doi: 10.1186/s12864-020-6708-8 (PMC7132880; doi:10.1186/s12864-020-6708-8)
Supplement: Supplementary file 1 — Additional file 1 Table S1. List of primer sequences. Fig. S1. Phylogenetic analysis of 5 MIOX proteins from S. lycopersicum with other species MIOX proteins. Fig. S2. Alignment of 5 MIOX protein sequence from S. lycopersicum. Multiple sequence alignments were conducted using ClustalW2. Fig. S3. Chromosomal distribution of the SlMIOX genes. A total of 5 SlMIOX genes were mapped onto tomato chromosomes. The gene identity numbers are provided, and respective chromosome numbers are shown at the top. [file 12864_2020_6708_MOESM1_ESM.docx]

**Supplementary material**

**Genome-wide analysis of Myo-inositol oxygenase gene family in tomato reveals their involvement in ascorbic acid accumulation**

Shoaib Munir^1^, Muhammad Ali Mumtaz^1^, John Kojo Ahiakpa^1^, Genzhong Liu^1^, Weifang Chen^1^, Guolin Zhou^2^, Wei Zheng^3^, Zhibiao Ye^1, 3^, and Yuyang Zhang ^1, 3^*

^1^ Key Laboratory of Horticultural Plant Biology, Ministry of Education, Huazhong Agricultural University, Wuhan 430070, China

^2^ Wuhan Academy of Agricultural Sciences, Wuhan 430065, China

^3^ Huazhong Agricultural University Chuwei Institute of Advanced Seeds, Wuhan 430070, China

*Correspondence: Yuyang Zhang; Tel: +86 27 87283180; Fax: +86 27 87282010; Email: [yyzhang@mail.hzau.edu.cn](mailto:yyzhang@mail.hzau.edu.cn)

**Table S1.** List of primer sequences.

| **Additional file 1. Primers used for real-time RT-PCR.** | |  |  |  |
| --- | --- | --- | --- | --- |
| Gene | Full name | Accession No. | Forward primer (5'-3') | Reverse primer (5'-3') |
| *SlMIOX4* | myo-inositol oxygenase gene | Solyc12g008650 | GAGAAAATGACTATTCTCATTGAGCAGCC | CCATGGATTCAATTACCACCTCAGC |
| *GPI* | glucose-6-phosphate isomerase | Solyc04g076090 | TGCTCTTCAAAAGCGTGTCC | CGGCAATAAGTGCTCTGTCA |
| *PMI* | phosphomannose isomerase | Solyc02g086090 | TACATTGTGGTGGAACGAGGA | ACCCCATTTGGCAAGAACAG |
| *PMM* | phosphomannomutase | Solyc05g048760 | TTTACCCTCCATTACATTGCTGA | TCTTCTTGACTACAGTTTCTCCCA |
| *GMP1* | GDP-D-mannose pyrophosphorylase1 | Solyc03g096730 | AAACCTGAAATCGTGATGTGAGA | TGAAGAAGAGGAGAACTGGAAAC |
| *GMP2* | GDP-D-mannose pyrophosphorylase 2 | Solyc06g051270 | TTGGACCAGATGTTGCGATA | CATTGTCCAACGGTTGAGTG |
| *GMP3* | GMP synthase | Solyc07g006090 | AGATGAGGTGTGGGAAGTGC | TGCAGCTTCTGCAAATTCAC |
| *GMP4* | GDP-Man 4 6-dehydratase | Solyc03g118270 | AAGATCGGGCTACAGAGCAA | GAACCAAATGCCTCTTCCAA |
| *GME1* | GDP-Mannose 3′,5′-epimerase1 | Solyc01g097340 | AATCCGACTTCCGTGAGCC | CTGAGTTGCGACCACGGAC |
| *GME2* | GDP-Mannose 3′,5′-epimerase2 | Solyc04g077020 | CCATCACATTCCAGGACCAGA | CGTAATCCTCAACCCATCCTT |
| *GGP* | GDP-L-galactose-1-phosphate phosphorylase1 | Solyc06g073320 | GAAATCTGGTCTGTTCCTCTGTGA | TTCACACACCAACTCCACATTACA |
| *GPP* | L-galactose-1-phosphate phosphatase1 | Solyc04g014800 | AGCCGCTACAAACCCTCATCT | TGTCCGCTTTCCATCTCCTAT |
| *GalDH* | L-galactose dehydrogenase | Solyc01g106450 | CTTCTTACTGAGGCTGGTGGTC | AACCTCTTTAACAGACTTCATCCC |
| *GLDH* | L-galactono-1,4-lactone dehydrogenase | Solyc10g079470 | ATTGAGGTTCCCAAGGACATAG | ATGTTATTAGATAGGATGCGGTTT |
| *MDHAR* | monodehydroascorbate reductase | Solyc09g009390 | GGTGATGTTGCCACTTTTCCTTT | CGACAGACTTCCCTTGCTCACT |
| *DHAR* | dehydroascorbate reductase | Solyc05g054760 | CCTACCTTCGTCTCATTTCCG | TGAACAAACATTCTGCCCATT |
| *AO* | ascorbate oxidase | Solyc04g054690 | AGGATGGCTCAGAGTGTT | ATCAGGTAAGGCGTATGG |
| *cAPX* | Cytosolic ascorbate peroxidase | Solyc06g005150 | TGGAGCCCATTAGGGAGCA | GCCAGGGTGAAAGGGAACAT |
| *tAPX* | thylakoid ascorbate peroxidase | Solyc11g018550 | CTTTCTTCAATGGCTTCTCTCACCG | CAACCTGGTAGCGAAACACATGGG |
| *AOBP* | ascorbate oxidase promoter-binding protein | Solyc06g69760 | GCTTTCTCCCATTGAACTCCAG | GCCAGCCACTTGCTCTTATTGT |
| *ß-Actin* | Solanum lycopersicum Actin | Solyc04g011500 | ATGGCAGACGGAGAGGATATTCA | AGACGGAGAATGGCATGTGG |
| *Actin* | *Actin* | Solyc11g005330 | GTCCTCTTCCAGCCATCCA | ACCACTGAGCACAATGTTACCG |

**Supplementary Figures legend**

**Fig. S1.**Phylogenetic analysis of 5 *MIOX* proteins from *S. lycopersicum* with other species MIOX proteins.

**Fig. S2.** Alignment of 5 *MIOX* protein sequence from *S. lycopersicum*. Multiple sequence alignments were conducted using ClustalW2.

**Figure S3**. Chromosomal distribution of the *SlMIOX* genes. A total of 5 *SlMIOX* genes were mapped onto tomato chromosomes. The gene identity numbers are provided and respective chromosome numbers are shown at the top.

**
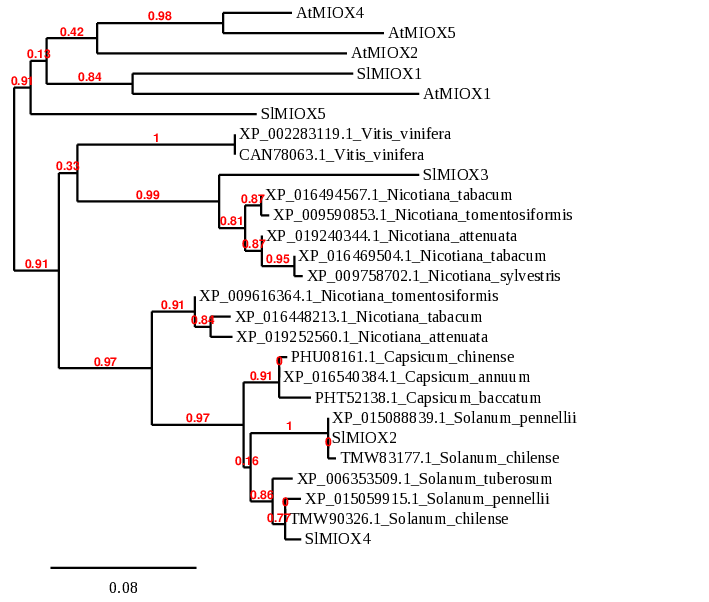
Figure S1**

**Figure S2**

**SlMIOX2** MAIVQALFPSQYLCMNKTKNNTQIGSKRSIQVSCRSSNQEDLLSRRQTSYNHSDEFGLQV

**SlMIOX4** --------------MTILIEQPEFGSQ---------------VEEKKVSFN-ANELILDG

**SlMIOX3** --------------MTFLVAQT------------------------------------DH

**SlMIOX1** -------MRQVGPVLGRRKEECEIFDFS--------------SVFSFTMTILIEQLPVEI

**SlMIOX5** ---------------------------------------------------------MTI

**SlMIOX2** EVEEKKVASFNGNE---GVVFEVSNINAFGQSFRDYN-AKSERQKSVEEFYRVQHINQTY

**SlMIOX4** GFMVPKTLSSQDE------IFEVPDINAFGQSFRDYN-VESERQKSVEEFYRVQHINQTY

**SlMIOX3** ELDNKKAIS--DG------IFVVPGNNAFGNSFRDYN-AEAERQKIVRELYRQSHINQTY

**SlMIOX1** QADETNIHAQNQKELVLGDGFAVPETNAFGHNFRDYT-VESARQQGVENFYKINHFNQTY

**SlMIOX5** QLEGKKIQDQDQ--------FMVPQTNAFGHDFRDYNNAQSERQKGVEKFYKTQHINQTY

: * *. ****:.****. .:: **: *.::*: .*:****

**SlMIOX2** DYVTKMRKEYGKLNKIEMSIWDCCELLNDVVDDSDPDLDEPQIEHLLQTAEAIRKDYPNE

**SlMIOX4** DYVKKMRKEYGKLNKIEMSIWDCCELLNDVVDDSDPDLDEPQIEHLLQTAEAIRKDYPNE

**SlMIOX3** DFVKKMREEYGKMNKVEMSIWECCELLNEVVDDSDPDLDEPQIEHLLQTAEAIRKDYPNE

**SlMIOX1** DYVSKMRVEYAKLDKAEMSIWECCELLNDVVDDSDPDLDEPQIQHLLQSAEAIRKDYPDE

**SlMIOX5** DFVKKMREDYIKLDKAKMSIWECCELLNDVVDDSDPDLDEPQIQHLLQSAEAIRKDYPNE

*:*.*** :* *::* :****:******:**************:****:*********:*

**SlMIOX2** DWLHLTGLIHDLGKVLLHPSFGELPQWAVVGDTFPLGCAFDESIVHHKYFKENQDINNMI

**SlMIOX4** DWLHLTGLIHDLGKVLLHPSFGGLPQWAVVGDTFPLGCAFDESIVHHKYFKENPDINNNI

**SlMIOX3** DWLHLTGLIHDLGKVLLLPSFGGLPQWAVVGDTFPLGCAFDESIVLHEQLKGNPDNNNPT

**SlMIOX1** DWLHLTALIHDLGKVLLLPSFGELPQWAVVGDTFPVGCAFHDSIVHSKYFKENPDYNNTA

**SlMIOX5** DWLHLTALIHDLGKILVLPKFGGLPQWAVVGDTFPLGCAFDESNIHHKYFKENQDFNNPI

******.*******:*: *.** ************:****.:* : : :* * * **

**SlMIOX2** YNTKNGVYEEGCGLNKVVMSWGHDDYMYLVAKENGTTLPYAALFVIRYHSFYALHKSGAY

**SlMIOX4** YNTKNGVYEEGCGLDKVVMSWGHDDYMYLIAKENKTTLPSAALFVIRYHSFYALHRSGAY

**SlMIOX3** YNTKYGVYSEGCGLNNVVMSWGHDDYMYLVAKANKTTLPSAALFIIRYHSFYRKQY----

**SlMIOX1** YKSKFGVYSEGCGLDKVLMSWGHDDYMYLVAKENGTTLPSAGLFIIRYHSFYALHKSGAY

**SlMIOX5** YNTKNGIYNDSIGLENVMMSWGHDDYMYMVAKENGTTLPSAGLFIIRYHSFYPLHKNGGY

*::* *:*.:. **::*:**********::** * **** *.**:******* :

**SlMIOX2** KHLMNEEDKENIKWLNIFNKYDLYSKSSVRIDVENVKPYYLSLIQKYFPKILRW

**SlMIOX4** THLMNEEDKENMKWLNIFNKYDLYSKSKVRIDVEKVKPYYLSLIEKYFPTKLRW

**SlMIOX3** ----------------IINSFFLFSTT-----------YLL-------------

**SlMIOX1** KELMNEEDKENLKWLHIFNKYDLYSKSKVQVNVEEVKPYYMSLIEKYFPAKLKW

**SlMIOX5** KHLMNDEDEENLKWLHVFNKYDLYSKSKVHVNVEEVKPYYMSLIEKYFPAKLRW

::*.: *:*.: * :


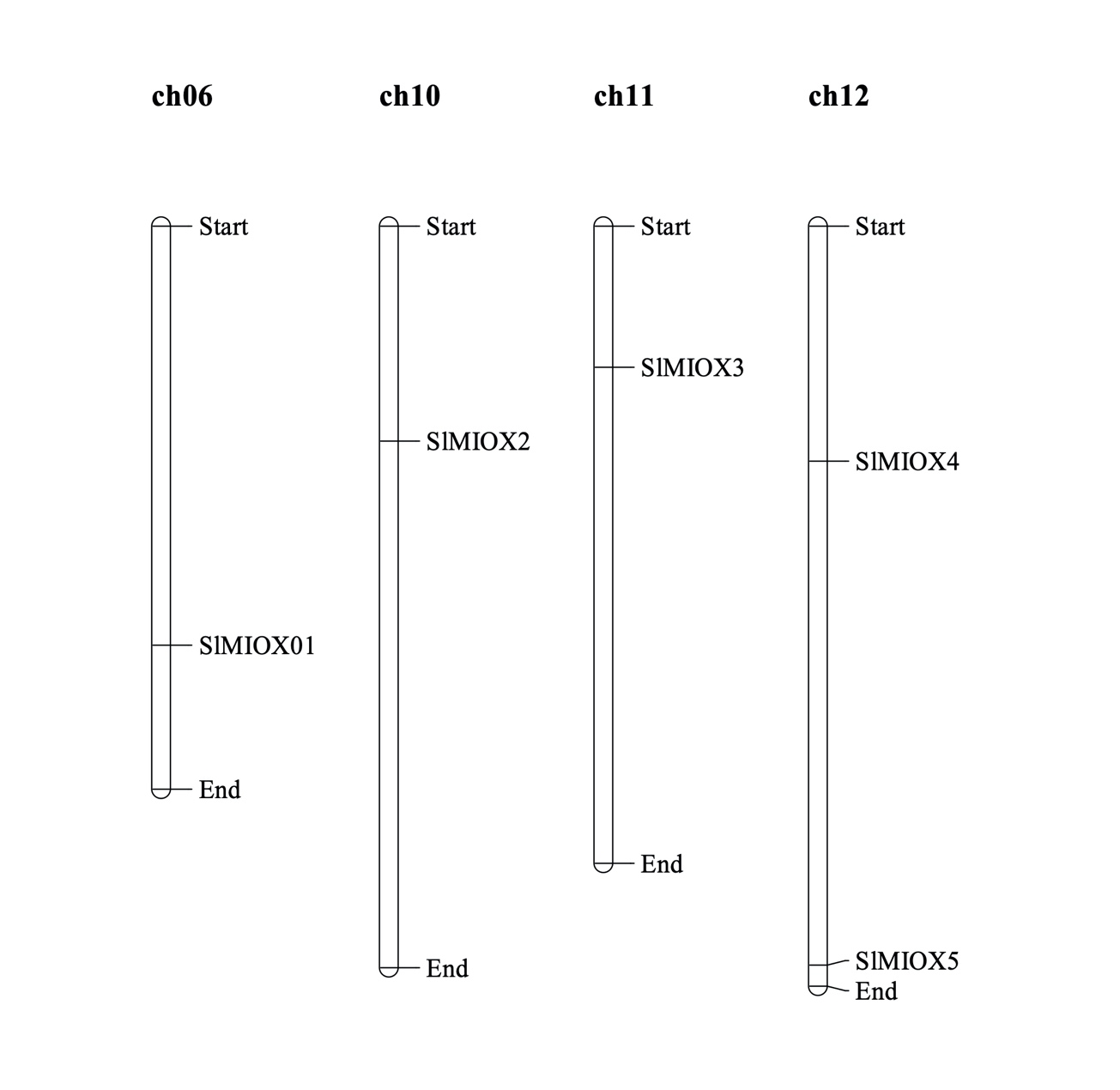
**Figure S3**
